# Plastome Phylogenomics of *Aucuba* (Garryaceae)

**DOI:** 10.3389/fgene.2022.753719

**Published:** 2022-01-24

**Authors:** Yuan Huang, Linyuan Fan, Jian Huang, Guohua Zhou, Xiong Chen, Jiahui Chen

**Affiliations:** ^1^ School of Life Sciences, Yunnan Normal University, Kunming, China; ^2^ Yunnan General Administration of Foresty Seeds and Seedlings, Kunming, China; ^3^ Chinese Medicinal Resources Co. LTD, Yunnan Baiyao Group, Kunming, China; ^4^ CAS Key Laboratory for Plant Diversity and Biogeography of East Asia, Kunming Institute of Botany, Chinese Academy of Sciences, Kunming, China

**Keywords:** Garryales, Garryaceae, *Aucuba*, phylogenomics, molecular dating

## Abstract

*Aucuba* (Garryaceae), which includes approximately ten evergreen woody species, is a genus endemic to East Asia. Their striking morphological features give *Aucuba* species remarkable ornamental value. Owing to high levels of morphological divergence and plasticity, species definitions of *Aucuba* remain perplexing and problematic. Here, we sequenced and characterized the complete plastid genomes (plastomes) of three *Aucuba* species: *Aucuba chlorascens*, *Aucuba eriobotryifolia*, and *Aucuba japonica*. Incorporating *Aucuba* plastomes available in GenBank, a total of seven *Aucuba* plastomes, representing six out of ten species of *Aucuba*, were used for comparative plastome analysis, phylogenetic analysis and divergence time estimation in this study. Comparative analyses revealed that plastomes of *Aucuba* are highly conserved in size, structure, gene content, and organization, and exhibit high levels of sequence similarity. Phylogenetic reconstruction based on 68 plastid protein-coding genes strongly supported the monophyly of Garryales, Garryaceae and *Aucuba. Aucuba eriobotryifolia* was sister to the other *Aucuba* species examined, consistent with its unique fused anther locule. The divergence time of *Aucuba* was estimated to be approximately late Miocene. Extant *Aucuba* species derived from recent divergence events associated with the establishment of monsoonal climates in East Asia and climatic fluctuations.

## Introduction

*Aucuba* Thunberg is a small genus of 10 evergreen woody species endemic to East Asia distributed in the Eastern Himalayas, China, Korea, Japan, Myanmar, and Vietnam (Xiang and Boufford, 2005). *Aucuba* is easy to recognize owing to its morphological distinctiveness. However, the morphologies of *Aucuba* species are highly divergent and plastic, making morphology-based taxonomy perplexing and problematic (Xiang and Boufford, 2005) and hindering the effective conservation and exploitation of the germplasm. The taxonomic affinities of *Aucuba* have been in dispute since the establishment of the genus. Historically, this genus was placed into either Cornaceae (Harms, 1898; Wangerin, 1910; Hutchinson, 1967; Cronquist, 1988) or the monotypic family Aucubaceae (Willis and Shaw, 1973; Takhtajan, 1980; Bremer et al., 1998). Recently, phylogenetic analyses based on chloroplast DNA sequences revealed a sister relationship between *Aucuba* and *Garrya*, and the two genera are in turn closely related to Eucommiaceae (Xiang et al., 1993; Xiang and Soltis, 1998; Soltis et al., 2000; Bremer et al., 2003; Stull et al., 2015). Since *Aucuba* and *Garrya* show high levels of similarity in their morphologies and chemical components, they were grouped in Garryaceae. Together with the monotypic Eucommiaceae, which includes only one species (*Eucommia ulmoides*), Garryaceae was placed in the order Garryales (Bremer et al., 2003).

*Aucuba* possesses remarkable horticultural merits. Because of their evergreen habit, spotted and colorful leaves, and showy fruits, *Aucuba* species have been widely introduced and cultivated as garden plants for centuries in East Asia, Europe, and North America (Hagedoorn, 1950). Previous research on *Aucuba* mainly focused on cultivation management, introduction and domestication, phytochemistry, and cytogenetics (Hagedoorn, 1950; Allen, 1990; Ohi et al., 2003; Lehrer, 2009). Genomic resources are crucial for plant breeding; however, they have received little attention. Plastome-based phylogenomics would provide more convincing evidence in the *Aucuba phylogeny.*

Chloroplasts are organelles in green plants that perform photosynthesis and the biosynthesis of starch, fatty acids, pigments, and amino acids (Daniell et al., 2016). Gene content, structural arrangement, gene loss or pseudogenization, cytonuclear gene transfer, and sequence variations can provide informative and valuable resources for elucidating evolutionary relationships and species discrimination (Jansen et al., 2007; Moore et al., 2007). With the improvement of next-generation DNA sequencing, plastome sequencing has been widely used in recent years to investigate evolutionary relationships among closely related species (Jansen et al., 2007; Barrett et al., 2016). The availability and use of complete plastome sequences in biotechnology is likely to increase the performance of cultivated plants in the field (Rogalski et al., 2015; Daniell et al., 2016).

Here, based on plastomes of seven *Aucuba* taxa (three species, i.e., *Aucuba chlorascens*, *A. eriobotryifolia*, and *A. japonica,* were newly sequenced in this study), we performed phylogenomic analyses and fossil-calibrated molecular dating to 1) elucidate the relationships of Garryales; 2) investigate interspecific relationships within *Aucuba*; and 3) infer the history of species diversification for *Aucuba.* The plastid genomic resources presented here will be beneficial for the conservation and exploitation of *Aucuba* species.

## Results

### General Features and Higher Variable of Aucuba Plastomes

Paired-end Illumina sequencing generated over 30 million clean reads for each species. *De novo* assembly yielded three complete *Aucuba* plastomes, each identically encoding 114 unique genes: 80 protein-coding genes, 30 tRNAs, and 4 rDNA, which is the same as the other four *Aucuba* plastomes downloaded from GenBank (Table 1). The plastome size ranged from 158084 bp to 158,237 bp (Table 1).

**TABLE 1:** Features of *Aucuba* plastomes.

		*A. chlorascens* ^a^	*A. eriobotryifolia* ^a^	*A. japonica* ^a^	*A. japonica* var. *variegata* ^b^	*A. chinensis* ^b^	*A. himalaica* ^b^	*A. obcordata* ^b^
GenBank Accession		MT338539	MT338540	MT338541	MW556466	MW800961	MW801214	NC_056113
Sequencing coverage (×)		288.577	345.936	536.433	/	/	/	/
Genome Size (bp)		158,084	158,113	158,237	158237	158196	158196	157993
Large single copy (bp)		87,518	87,281	87,505	87505	87486	87486	87322
Inverted repeats (bp)		26,008	26,143	26,094	26094	26088	26088	26094
Small single copy (bp)		18,550	18,546	18,544	18544	18534	18534	18483
Total number of genes		114	114	114	114	114	114	114
Coding genes (CDS)		80	80	80	80	80	80	80
Transfer RNA genes (tRNA)		30	30	30	30	30	30	30
Ribosomal RNA genes (rRNA)		4	4	4	4	4	4	4
GC content (%)	Overall	37.7	37.7	37.7	37.7	37.7	37.7	37.8
	LSC	35.9	35.9	35.9	35.9	35.9	35.9	35.9
	IR	43.1	43.0	43.0	43	43.1	43.1	43
	SSC	31.5	31.5	31.6	31.6	31.5	31.4	31.5

a Plastomes newly generated and assembled in this study.

b Plastomes downloaded from NCBI.

The newly generated *Aucuba* plastomes exhibited the typical quadripartite structure (Supplementary Figure S1), consisting of a pair of inverted regions (IRs) (26,008 to 26,143 bp in length) separated by a large single copy region (LSC) (87,281 to 87,505 bp in length) region and a small signle copy region (SSC) (18,544 to 18,550 bp in length) region (Table 1 **)**. The overall GC content among these *Aucuba* plastomes was similar and was unevenly distributed in LSC, SSC, and IRs (Table 1).

**FIGURE 1:**
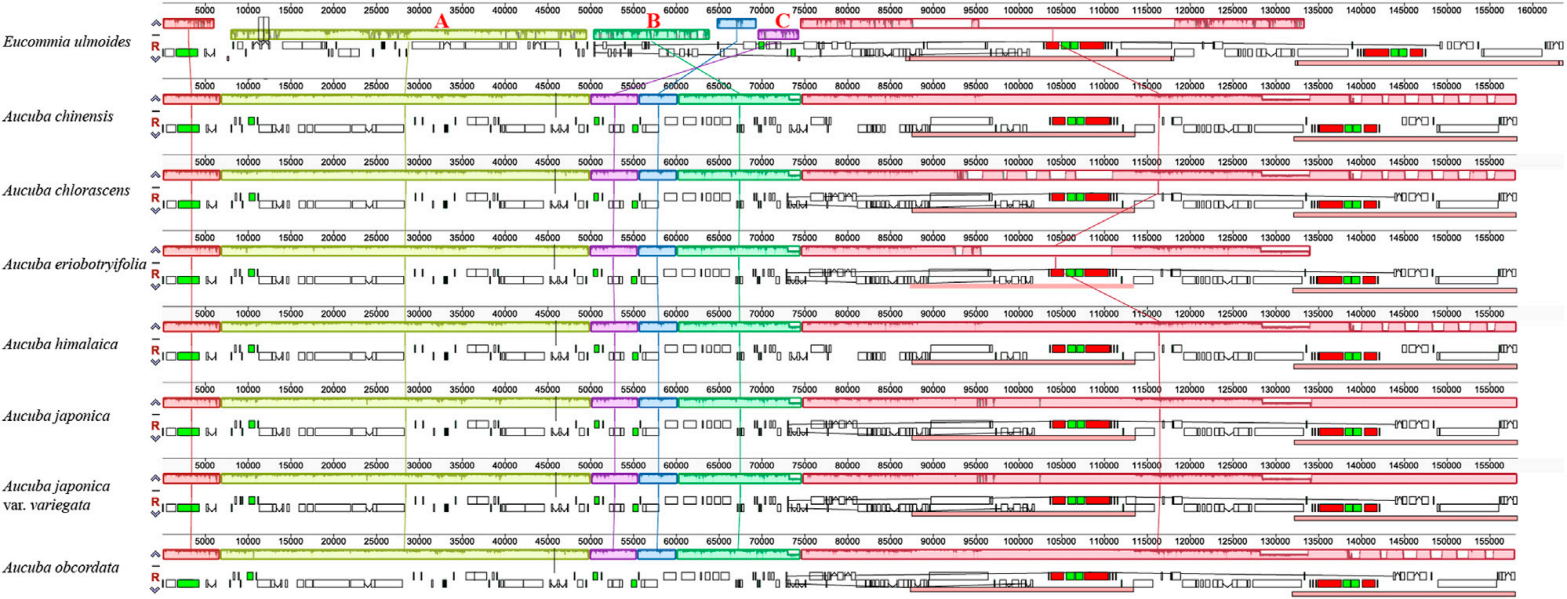
Genomic rearrangements detected in *Eucommia ulmoides* when compared with plastomes of *Aucuba*.

**FIGURE 2:**
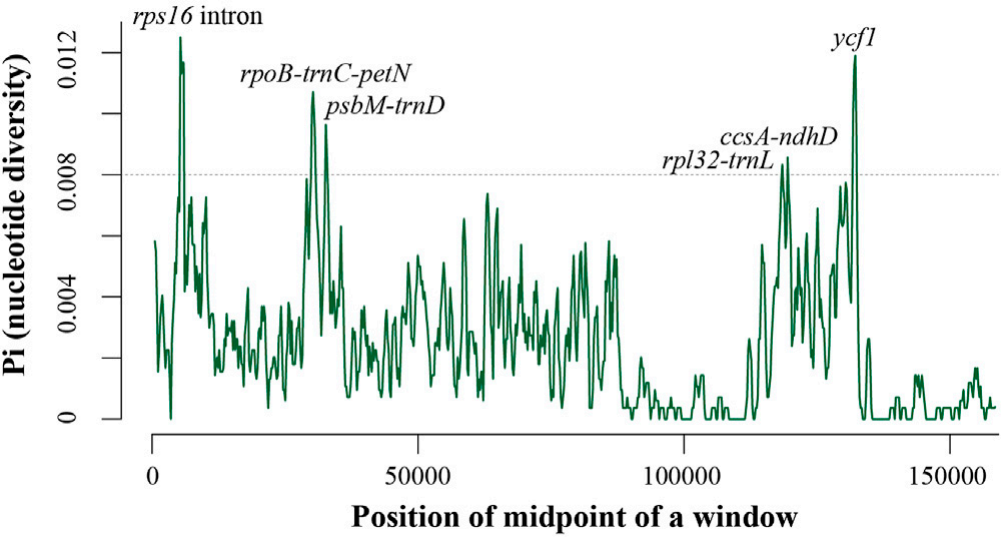
Nucleotide diversity (Pi) in the complete plastomes of seven *Aucuba* plastomes. Sliding window analysis with a window length of 800 bp and a step size of 200 bp.

We found that the length, structure, gene content, and organization of *Aucuba* plastomes are highly conserved, and exhibit high levels of sequence similarity (Figure 1, Supplementary Figure S2, Table 1). Nevertheless, sliding window analysis of entire plastomes revealed six plastid DNA regions with relatively higher nucleotide diversity (Pi > 0.008) in *Aucuba* (Figure 2), namely, *rps16* intron, *rpoB-trnC-petN*, *psbM-trnD*, *rpl32-trnL*, *ccsA-ndhD*, and *ycf1*.

### Garryales Plastome Rearrangements and Synteny

We identified three large inversions and an infragenomic translocation within the *E. ulmoides* plastome when compared to *Aucuba* plastomes (Figure 1), including an inversion of ∼46 kb from *rps16* to *trnT_UGU* (A), an inversion of ∼17 kb between *trnQ_UUG* and *rps12* (B), and an inversion of ∼6 kb located between *trnL_UAA* and *trnV_UAC* (C). The latter two sequence regions exchanged positions with each other. In the genus *Aucuba*, we found that the structure and synteny of the plastomes of the seven *Aucuba* taxa are highly conserved (Figure 1).

### Phylogenetic Relationships and Divergence Time Estimation

**FIGURE 3:**
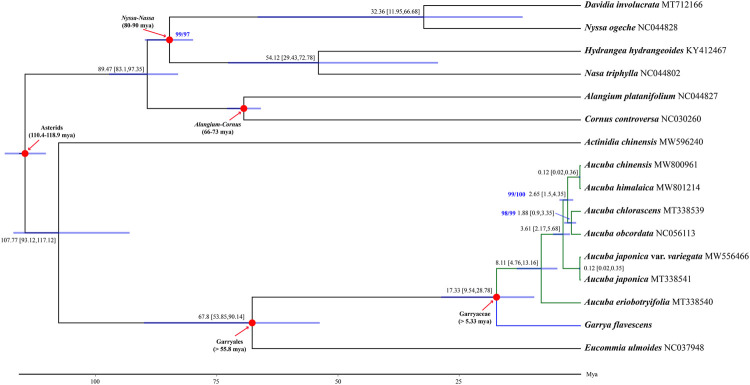
A chronogram based on 68 plastid CDSs of 16 asterids illustrating divergence times of *Aucuba*. Mean divergence times and their 95% posterior probability intervals are shown near each node. The blue bars at the nodes indicate 95% posterior probability intervals. Numbers highlighted in blue near the clades are support values reported as SH-aLRT (%)/UFBoot (%), and only values that are not 100/100 are shown. The GenBank accession number for each taxon was followed by taxa name. Red arrows show the calibrating clades for molecular dating, time used for calibrating are also shown. Divergence time and the timeline are indicated in million years ago (Mya).

Phylogenetic reconstruction based on 68 coding DNA sequences (CDSs) revealed that Garryales, Garryaceae and *Aucuba* were recovered as robust monophyletic clades (SH-alRT/UFBoot = 100/100), with *Eucommia* sister to Garryaceae. Within *Aucuba*, *A. eriobotryifolia* is sister to the other five *Aucuba* species. *A. chinensis* clustered with *A. himalaica*, and this clade was sister to a clade consisting of *A. chlorascens* and *A. obcordata*. The above species was further sister to *A. japonica* (Figure 3).

Fossil-calibrated molecular dating indicated that the crown age of Garryaceae is approximately 17.33 Mya [95% highest posterior density (HPD): 9.54–28.78 Mya], and the diversification of extant *Aucuba* species initiated at approximately 8.11 Mya [95% HPD: 4.76–13.16 Mya], which is in the late Miocene. The diversification of the major *Aucuba* (except *Aucuba eriobotryifolia* in the studied *Aucuba* species) lineage occurred at approximately 3.61 Mya [95% HPD: 2.17–5.68 Mya], in the middle Pliocene. The other *Aucuba* clades diverged between approximately 0.12–2.65 Mya (Figure 3).

## Discussion

### Plastome Comparison

The sizes of *Aucuba* plastomes reported in this study fall with the average size of angiosperm plastomes (Wicke et al., 2011). We found that the plastomes of *Aucuba* species are highly conserved in terms of genome synteny, structure and gene number. Large rearrangements in the LSC regions suggest that Garryales plastomes are highly divergent in gene organization despite exhibiting high levels of similarity in gene content. Interestingly, the inversions observed in the LSC of the *E. ulmoides* plastome were located flanking the LSC and near the LSC/IR junctions. The IR region of *E. ulmoides* (30535 bp) was significantly larger than that of *Aucuba* species (∼26000 bp). This supports the idea that inversions in plastomes might be linked to IR expansion/contraction (Bruneau et al., 1990). Moreover, the regions flanking the inversions contained tRNAs, coinciding with the assumption that tRNA activity most likely triggers inversions in plastomes (Walker et al., 2014).

High sequence similarity among the seven *Aucuba* plastomes indicates that few sequence variations have accumulated since the divergence of these species. The diversity and plasticity of morphological characteristics among *Aucuba* species has led to difficulties reconstructing their taxonomy. Plastid DNA sequences *rbcL*, *matK*, and *psbA*-*trnH* are recommended as standard DNA barcodes for plant species discrimination (Hollingsworth et al., 2011); our analysis analyses revealed that these sequences exhibit relatively low levels of variation among *Aucuba* species (Supplementary Figure S2). These standard DNA barcodes therefore have limited discriminatory power in *Aucuba*. The complete plastome DNA sequences analyzed in this study provide genomic resources for the development of novel DNA barcodes. Based on plastome-wide analysis of sequence variability, we propose six plastid DNA regions harboring relatively high proportions of variable sites. These sequences can serve as potential effective DNA barcodes for species identification and germplasm genotyping in *Aucuba*. However, we did not sequence all *Aucuba* species (six out of ten), and only one individual for each studied taxon was sequenced. The effectiveness of these potential DNA barcodes needs further research and validation.

### Phylogenetic Inferences and Taxonomical Implications

Complete plastome sequences have been widely used for resolving recalcitrant relationships in phylogenetically challenging taxa (Jansen et al., 2007; Barrett et al., 2013; Stull et al., 2015). In this study, the phylogenetic placement of *Aucuba* was inferred by reconstructing phylogenetic relationships based on a large dataset comprising 68 plastid CDSs. Our data strongly support the sister relationship between *E. ulmoides* and *Aucuba*, as well as the monophyly of both Garryales and Garryaceae. This result is consistent with previous molecular phylogenetic analyses (Xiang et al., 1993; Xiang and Soltis, 1998; Soltis et al., 2000; Bremer et al., 2003; Stull et al., 2015), providing plastid phylogenomic evidence to accept the order Garryales as circumscribed by Bremer et al. (2003).

Within *Aucuba*, *A. eriobotryfolia* was sister to the other five species, which is consistent with one of its unique traits: anthers of *A. eriobotryfolia* fused into one locule, which differs from other *Aucuba* species, which have two locules (Supplementary Figure S3). This indicates that anther locule number in *Aucuba* might be a key character in the evolution of *Aucuba*. For other clades of *Aucuba* revealed by our phylogeny, there were no obvious supporting morphological features. Some species of *Aucuba* are difficult to distinguish from each other, as the characters used to separate them are combinations of many quantitative traits (e.g., leaf length, number of leaf teeth, petiole length, density of hairs) and traits that may variable (e.g., leaf shap, leaf margin serrate or not). Identification of *Aucuba* species needs consider all of these variable traits. Stable reproductive relate traits of *Aucuba* used for species determination are rare, staminate inflorescences type, i.e., paniculate or racemose-paniculate, is another traits beside locule number. However, staminate inflorescence type was not accord with toplogical structure of *Aucuba* (Supplementary Figure S3). and leaf serration, that are widely used in the identification of *Aucuba* species.

### Recent Species Divergence in *Aucuba*

The divergence time of *Aucuba* was estimated at 8.11 Mya, around the late Miocene, involving the speciation of *A. eriobotryifolia*. The most extensive species divergence events in *Aucuba*, resulting in the divergence of the remaining five extant species examined, occurred in the middle Pliocene (3.61 Mya). The Asian monsoon has increasingly intensified since the Miocene, established a humid climate and resulted in the expansion of forests in East Asia (Yao et al., 2011). Pronounced wet/dry climatic fluctuations have occurred since the late Miocene (∼7 Mya) and have been even more intense since the late Pliocene. In approximately the same period, other paleoclimatic events included Miocene cooling and central Asia aridification (reviewed by Favre et al. (2015), Muellner-Riehl (2019). Climatic fluctuations, including wet/dry glaciation/interglaciation cycles and temperature fluctuations, could result in dramatic contraction/expansion of species ranges in the Northern Hemisphere (He et al., 2018; Abbott, 2019). In addition, the increased complexity of topography in East Asia, which might have blocked regional gene flow and boosted vicariance (Favre et al., 2015), is believed to have triggered rapid speciation in many plant lineages in East Asia (Favre et al., 2015; Muellner-Riehl, 2019). Similarly, these events would have triggered species radiation in *Aucuba* and hampered their dispersal to central Asia because of Asian aridification since the late early Miocene (reviewed by Muellner-Riehl, 2019) and therefore restricted the distribution area of *Aucuba* in East Asia.

## Materials and Methods

### DNA Extraction, Shotgun Sequencing, Plastome Assembly, and Annotation

Samples of *Aucuba chlorascens*, *A. eriobotryifolia*, and *A. japonica* were collected from the Botanical Garden of Kunming Institute of Botany, Kunming, China. The formal identification of the plant material was undertaken by the Herbarium of Kunming Institute of Botany (KUN), and voucher specimens were deposited at KUN (JC-YJ-64, JC-YJ-66, JC-YJ-68). Genomic DNA was isolated from ∼50 mg silica-gel-dried leaf tissues using the CTAB method (Doyle and Doyle, 1987). Genomic DNA was fragmented into 500 bp fragments by ultrasonic disruption to construct libraries. Paired-end libraries were prepared according to the manufacturer’s protocol (Illumina, San Diego, CA, United States) for sequencing on the Illumina HiSeq 2500 system.

Low-quality reads were removed from raw data using NGS QC Toolkit (Patel et al., 2012) by setting the cutoff value for percentage of read length to 80 and PHRED quality scores to 30. Filtered reads were used for *de novo* assembly of *Aucuba* plastomes using NOVOPlasty v2.7.0 (Dierckxsens et al., 2017), setting the k-mer size to 30. The *rbcL* CDS of *A. japonica* (GenBank Accession: AY725858) was used as a seed, which is required for NOVOPlasty software to assembly complete plastomes by iterative extension. Assembled plastomes were annotated using GeSeq (Tillich et al., 2017). Incorrect start codons and premature stop codons were corrected manually, and incorrect intron/exon boundaries for CDS were corrected manually by comparing with close relate plastome of *Eucommia ulmoides* (GenBank accession: KU204775). Annotated tRNA genes were further verified using tRNAscan-SE 1.21 (Schattner et al., 2005) with default parameters. Annotated plastomes were illustrated using the online program OrganellarGenomeDRAW (Lohse et al., 2007).

### Comparison of Plastomes

The whole plastome DNA sequence of *Eucommia ulmoides* (GenBank accession: KU204775) was used as a reference. To investigate differences in the Garryales plastomes, we progressively aligned the *E. ulmoides* with our assembled *Aucuba* and four other *Aucuba* plastomes downloaded from NCBI, i.e., *Aucuba chinenesis*, *Aucuba himalaica*, *Aucuba japonica* var. *variegata*, *Aucuba obcordata*, using the multiple genome alignment software Mauve 2.3.1 (Darling et al., 2010) with default parameters. The plastomes of *Aucuba* were pairwise aligned using the mVISTA program (https://genome.lbl.gov/vista) in LAGAN mode. Nucleotide diversity (Pi) among *Aucuba* plastomes was calculated using DnaSP 5.10.01 (Librado and Rozas, 2009). The step size was set to 200 bp, with an 800 bp window length.

### Phylogenetic Analyses and Divergence Time Estimation

Phylogenetic reconstruction included nine Garryales taxa, including seven taxa and six species of *Aucuba*, as well as *Garrya flavescens* (CDS of this species was downloaded from NCBI as reported by Stull et al. (2015) and *Eucommia ulmoides*, of which three *Aucuba* plastomes were newly generated in the present study. To investigate the phylogenetic relationships and divergence time of Garryales and Garryaceae, the complete plastomes of an additional seven taxa from the Asterids representing clades with credible fossil records were downloaded from NCBI and included in the analyses (Supplementary Table S1). We reannotated the plastomes obtained from NCBI using GeSeq (Tillich et al., 2017). Sixty-eight CDSs (see Supplementary Table S2 for sequence information) commonly shared by these taxa were used for phylogenetic reconstruction and divergence time estimation. Alignments of these genes were concatenated using MAFFT v7.475 software (Katoh and Standley, 2013).

We evaluated the best-fit model of evolution for each CDS with the minimum Bayesian information criterion score computed by ModelFinder (Kalyaanamoorthy et al., 2017). Phylogenetic inference was conducted by maximum likelihood (ML) using IQ-TREE v2.1.3 (Minh et al., 2020), parameters were estimated separately for each CDS using an edge-linked proportional partition model with separate substitution models and separate rates across sites (Chernomor et al., 2016). The best-fitted models are listed in Supplementary Table S2. The ML tree was inferred independently 20 times, and the best-known ML tree with the highest log-likelihood was selected. SH-like approximate likelihood ratio test (SH-aLRT) (Guindon et al., 2010) and ultrafast bootstrap (UFBoot) (Hoang et al., 2018) support values were calculated from 5000 replicates with IQ-TREE v2.1.3.

Molecular clock estimation was based on the ML topology generated above. We used four calibration points based on credible macrofossils: the ages of the crown groups of the Alangium-Cornus and Nyssa-Nasa clades were calibrated to 66–73 and 80–90 million years ago (Mya), respectively (Schenk and Hufford, 2010; Xiang et al., 2011; Rose et al., 2018); the minimum crown ages of Garryaceae and Garryales were set to 5.33 and 55.8 Mya, respectively (Martínez-Millán, 2010; Manchester et al., 2015). In addition to fossils, we restricted the crown age of asterids using secondary calibration points from Magallon et al. (2015). Divergence times were estimated under a relaxed molecular clock model by using the MCMCTree of the PAML 4.9a package (Yang, 2007) with an independent substitution rate, and samples were drawn every 10 iterations until completion of 10^7^ iterations. Overall, we ran 1.1 × 10^8^ iterations and discarded 10^7^ iterations as burn-in. To check for convergence to the stationary distribution, each analysis was run in duplicate, and the results were compared between runs.

## Data Availability

The data presented in the study are deposited in the National Center of Biotechnology Information (NCBI, https://www.ncbi.nlm.nih.gov/) repository, accession number MT338539, MT338540, MT338541.

## Supplementary Material

Supplementary Figure S1 Map of three *Aucuba* plastomes newly generated in this study.

Supplementary Figure S2 Alignment of *Aucuba* plastomes using mVISTA, showing the percentages of sequence identity (y-axis).

## References

[B1] Abbott R. J. (2019). A Mixing-Isolation-Mixing Model of Speciation Can Potentially Explain Hotspots of Species Diversity. Natl. Sci. Rev. 6, 290–291. 10.1093/nsr/nwy112 34691866 PMC8291541

[B2] Allen E. F. (1990). *Aucuba japonica*: an Example of Cytoplasmic Inheritance. The plantsman 11, 244–245. 10.1016/0167-8655(90)90062-7

[B3] Barrett C. F. Baker W. J. Comer J. R. Conran J. G. Lahmeyer S. C. Leebens‐Mack J. H. (2016). Plastid Genomes Reveal Support for Deep Phylogenetic Relationships and Extensive Rate Variation Among Palms and Other Commelinid Monocots. New Phytol. 209, 855–870. 10.1111/nph.13617 26350789

[B4] Barrett C. F. Davis J. I. Leebens-Mack J. Conran J. G. Stevenson D. W. (2013). Plastid Genomes and Deep Relationships Among the Commelinid Monocot Angiosperms. Cladistics 29, 65–87. 10.1111/j.1096-0031.2012.00418.x 34814372

[B5] Bremer B. Bremer K. Chase M. W. Reveal J. L. Soltis D. E. Soltis P. S. (2003). An Update of the Angiosperm Phylogeny Group Classification for the Orders and Families of Flowering Plants: APG II. Bot. J. Linn. Soc. 141, 399–436. 10.1046/j.1095-8339.2003.t01-1-00158.x

[B6] Bremer K. Chase M. W. Stevens P. F. Anderberg A. A. Backlund A. Bremer B. (1998). An Ordinal Classification for the Families of Flowering Plants. Ann. Mo. Bot. Garden 85, 531–553.

[B7] Bruneau A. Doyle J. J. Palmer J. D. (1990). A Chloroplast DNA Inversion as a Subtribal Character in the Phaseoleae (Leguminosae). Syst. Bot. 15, 378–386. 10.2307/2419351

[B8] Chernomor O. Von Haeseler A. Minh B. Q. (2016). Terrace Aware Data Structure for Phylogenomic Inference from Supermatrices. Syst. Biol. 65, 997–1008. 10.1093/sysbio/syw037 27121966 PMC5066062

[B9] Cronquist A. (1988). The Evolution and Classification of Flowering Plants. Bronx, N.Y., USA: New York Botanical Garden.

[B10] Daniell H. Lin C.-S. Yu M. Chang W.-J. (2016). Chloroplast Genomes: Diversity, Evolution, and Applications in Genetic Engineering. Genome Biol. 17, 134. 10.1186/s13059-016-1004-2 27339192 PMC4918201

[B46] Darling A. E. Mau B. Perna N. T. (2010). progressiveMauve: Multiple Genome Alignment With Gene Gain, Loss and Rearrangement. PLoS One 5, e11147. 10.1371/journal.pone.0011147 20593022 PMC2892488

[B47] Dierckxsens N. Mardulyn P. Smits G. (2017). NOVOPlasty: De Novo Assembly of Organelle Genomes from Whole Genome Data. Nucleic Acids Res. 45, e18. 10.1093/nar/gkw955 28204566 PMC5389512

[B48] Doyle J. J. Doyle J. L. (1987). A Rapid DNA Isolation Procedure for Small Quantities of Fresh Leaf Tissue. Phytochem. Bull. 19, 11–15. 10.1016/0031-9422(80)85004-7

[B11] Favre A. Päckert M. Pauls S. U. Jähnig S. C. Uhl D. Michalak I. (2015). The Role of the Uplift of the Qinghai‐Tibetan Plateau for the Evolution of Tibetan Biotas. Biol. Rev. 90, 236–253. 10.1111/brv.12107 24784793

[B12] Guindon S. Dufayard J.-F. Lefort V. Anisimova M. Hordijk W. Gascuel O. (2010). New Algorithms and Methods to Estimate Maximum-Likelihood Phylogenies: Assessing the Performance of PhyML 3.0. Syst. Biol. 59, 307–321. 10.1093/sysbio/syq010 20525638

[B13] Hagedoorn A. (1950). Plant Breeding. London: Crosby Lockwood & Son, Ltd.

[B14] Harms H. (1898). “Cornaceae,” in Nat. Pflanzenfam. Editors Engler A. Prantl K. (Leipzig: Wilhelm Engelmann), 250–270.

[B15] He Z. Li X. Yang M. Wang X. Zhong C. Duke N. C. (2018). Speciation with Gene Flow via Cycles of Isolation and Migration: Insights from Multiple Mangrove Taxa. Natl. Sci. Rev. 6, 275–288. 10.1093/nsr/nwy078 31258952 PMC6599600

[B16] Hoang D. T. Chernomor O. Von Haeseler A. Minh B. Q. Vinh L. S. (2018). UFBoot2: Improving the Ultrafast Bootstrap Approximation. Mol. Biol. Evol. 35, 518–522. 10.1093/molbev/msx281 29077904 PMC5850222

[B17] Hollingsworth P. M. Graham S. W. Little D. P. (2011). Choosing and Using a Plant DNA Barcode. PLOS ONE 6, e19254. 10.1371/journal.pone.0019254 21637336 PMC3102656

[B18] Hutchinson J. (1967). “Genera Plantarum,” in The Genera of Flowering Plants (Angiospermae). Editor H J (United Kingdom: Oxford University Press), 497–522.

[B19] Jansen R. K. Cai Z. Raubeson L. A. Daniell H. Depamphilis C. W. Leebens-Mack J. (2007). Analysis of 81 Genes from 64 Plastid Genomes Resolves Relationships in Angiosperms and Identifies Genome-Scale Evolutionary Patterns. Proc. Natl. Acad. Sci. 104, 19369–19374. 10.1073/pnas.0709121104 18048330 PMC2148296

[B20] Kalyaanamoorthy S. Minh B. Q. Wong T. K. F. Von Haeseler A. Jermiin L. S. (2017). ModelFinder: Fast Model Selection for Accurate Phylogenetic Estimates. Nat. Methods 14, 587–589. 10.1038/nmeth.4285 28481363 PMC5453245

[B21] Katoh K. Standley D. M. (2013). MAFFT Multiple Sequence Alignment Software Version 7: Improvements in Performance and Usability. Mol. Biol. Evol. 30, 772–780. 10.1093/molbev/mst010 23329690 PMC3603318

[B22] Lehrer J. (2009). Shedding New Light on Aucuba. Am. Nurseryman 209, 38.

[B49] Librado P. Rozas J. (2009). DnaSP v5: A Software for Comprehensive Analysis of DNA Polymorphism Data. Bioinformatics 25, 1451–1452. 10.1093/bioinformatics/btp187 19346325

[B50] Lohse M. Drechsel O. Bock R. (2007). OrganellarGenomeDRAW (OGDRAW): A Tool for the Easy Generation of High-Quality Custom Graphical Maps of Plastid and Mitochondrial Genomes. Curr. Genet. 52, 267–274. 10.1007/s00294-007-0161-y 17957369

[B23] Magallón S. Gómez‐Acevedo S. Sánchez‐Reyes L. L. Hernández‐Hernández T. (2015). A Metacalibrated Time‐tree Documents the Early Rise of Flowering Plant Phylogenetic Diversity. New Phytol. 207, 437–453. 10.1111/nph.13264 25615647

[B24] Manchester S. R. Grímsson F. Zetter R. (2015). Assessing the Fossil Record of Asterids in the Context of Our Current Phylogenetic Framework1. Ann. Mo. Bot. Garden 100, 329–363. 10.3417/2014033 PMC6485501 31031419

[B25] Martínez-Millán M. (2010). Fossil Record and Age of the Asteridae. Bot. Rev. 76, 83–135. 10.1007/s12229-010-9040-1

[B26] Minh B. Q. Schmidt H. A. Chernomor O. Schrempf D. Woodhams M. D. Von Haeseler A. (2020). IQ-TREE 2: New Models and Efficient Methods for Phylogenetic Inference in the Genomic Era. Mol. Biol. Evol. 37, 1530–1534. 10.1093/molbev/msaa015 32011700 PMC7182206

[B27] Moore M. J. Bell C. D. Soltis P. S. Soltis D. E. (2007). Using Plastid Genome-Scale Data to Resolve Enigmatic Relationships Among Basal Angiosperms. Proc. Natl. Acad. Sci. 104, 19363–19368. 10.1073/pnas.0708072104 18048334 PMC2148295

[B28] Muellner-Riehl A. N. (2019). Mountains as Evolutionary Arenas: Patterns, Emerging Approaches, Paradigm Shifts, and Their Implications for Plant Phylogeographic Research in the Tibeto-Himalayan Region. Front. Plant Sci. 10, 195. 10.3389/fpls.2019.00195 30936883 PMC6431670

[B29] Ohi T. Kajita T. Murata J. (2003). Distinct Geographic Structure as Evidenced by Chloroplast DNA Haplotypes and Ploidy Level in Japanese *Aucuba* (Aucubaceae). Am. J. Bot. 90, 1645–1652. 10.3732/ajb.90.11.1645 21653340

[B51] Patel R. K. Mukesh J. Liu Z. (2012). NGS QC Toolkit: A Toolkit for Quality Control of Next Generation Sequencing Data. PLoS One 7, e30619. 10.1371/journal.pone.0030619 22312429 PMC3270013

[B30] Rogalski M. do Nascimento Vieira L. Fraga H. P. Guerra M. P. (2015). Plastid Genomics in Horticultural Species: Importance and Applications for Plant Population Genetics, Evolution, and Biotechnology. Front. Plant Sci. 6, 586. 10.3389/fpls.2015.00586 26284102 PMC4520007

[B31] Rose J. P. Kleist T. J. Löfstrand S. D. Drew B. T. Schönenberger J. Sytsma K. J. (2018). Phylogeny, Historical Biogeography, and Diversification of Angiosperm Order Ericales Suggest Ancient Neotropical and East Asian Connections. Mol. Phylogenet. Evol. 122, 59–79. 10.1016/j.ympev.2018.01.014 29410353

[B52] Schattner P. Brooks A. N. Lowe T. M. (2005). The tRNAscan-SE, snoscan and snoGPS Web Servers for the Detection of tRNAs and snoRNAs. Nucleic Acids Res. 33, W686–W689. 10.1093/nar/gki366 15980563 PMC1160127

[B32] Schenk J. J. Hufford L. (2010). Effects of Substitution Models on Divergence Time Estimates: Simulations and an Empirical Study of Model Uncertainty Using Cornales. issn: 0363-6445 35, 578–592. 10.1600/036364410792495809

[B33] Soltis D. Soltis P. S. Chase M. W. Mort M. E. Albach D. C. Zanis M. J. (2000). Angiosperm Phylogeny Inferred from 18S rDNA, rbcL, and atpB Sequences. Bot. J. Linn. Soc. 133, 381–461. 10.1006/bojl.2000.0380

[B34] Stull G. W. Duno de Stefano R. Soltis D. E. Soltis P. S. (2015). Resolving Basal Lamiid Phylogeny and the Circumscription of Icacinaceae with a Plastome‐scale Data Set. Am. J. Bot. 102, 1794–1813. 10.3732/ajb.1500298 26507112

[B35] Takhtajan A. L. (1980). Outline of the Classification of Flowering Plants (Magnoliophyta). Bot. Rev. 46, 225–359. 10.1007/bf02861558

[B53] Tillich M. Lehwark P. Pellizzer T. Ulbricht-Jones E. S. Fischer A. Bock R. (2017). GeSeq - Versatile and Accurate Annotation of Organelle Genomes. Nucleic Acids Res. 45, W6–W11. 10.1093/nar/gkx391 28486635 PMC5570176

[B36] Walker J. F. Zanis M. J. Emery N. C. (2014). Comparative Analysis of Complete Chloroplast Genome Sequence and Inversion Variation in Lasthenia Burkei (Madieae, Asteraceae). Am. J. Bot. 101, 722–729. 10.3732/ajb.1400049 24699541

[B37] Wangerin W. (1910). “Cornaceae,” in Das Pflanzenreich IV. Editor Engler A. (Leipzig: H.F. Engelmann), 1–101.

[B38] Wicke S. Schneeweiss G. M. Depamphilis C. W. Müller K. F. Quandt D. (2011). The Evolution of the Plastid Chromosome in Land Plants: Gene Content, Gene Order, Gene Function. Plant Mol. Biol. 76, 273–297. 10.1007/s11103-011-9762-4 21424877 PMC3104136

[B39] Willis J. Shaw H. (1973). “Aucubaceae,” in A Dictionary of the Flowering Plants and Ferns. Editor Af I S (Cambridge: Cambridge University Press), 142–143.

[B40] Xiang Q.-Y. Soltis D. E. Morgan D. R. Soltis P. S. (1993). Phylogenetic Relationships of *Cornus* L. Sensu Lato and Putative Relatives Inferred from rbcL Sequence Data. Ann. Mo. Bot. Garden 80, 723–734. 10.2307/2399856

[B41] Xiang Q.-Y. Thomas D. T. Xiang Q. P. (2011). Resolving and Dating the Phylogeny of Cornales - Effects of Taxon Sampling, Data Partitions, and Fossil Calibrations. Mol. Phylogenet. Evol. 59, 123–138. 10.1016/j.ympev.2011.01.016 21300164

[B42] Xiang Q. Boufford D. (2005). “Aucuba,” in Flora of China. Editors Wu Z Y Hong D Y (Beijing & St Louis: Science Press), 222–226.

[B43] Xiang Q. Soltis D. (1998). “rbcL Sequence Data Define a Cornaceous Clade and Clarify Relationships of Cornaceae Sensu Lato,” in Sino-Japanese flora - its Characteristics and Diversification. Editor Boufford D E (Tokyo: Univ Mus Bull), 123–137.

[B44] Yang Z. (2007). PAML 4: Phylogenetic Analysis by Maximum Likelihood. Mol. Biol. Evol. 24, 1586–1591. 10.1093/molbev/msm088 17483113

[B45] Yao Y.-F. Bruch A. A. Mosbrugger V. Li C.-S. (2011). Quantitative Reconstruction of Miocene Climate Patterns and Evolution in Southern China Based on Plant Fossils. Palaeogeogr. Palaeoclimatol. Palaeoecol. 304, 291–307. 10.1016/j.palaeo.2010.04.012

