# Supplementary figures and images for: Plastome Phylogenomics of Aucuba (Garryaceae)
Source: Front Genet. 2022 Jan 24;13:753719. doi: 10.3389/fgene.2022.753719 (PMC8819091; doi:10.3389/fgene.2022.753719)

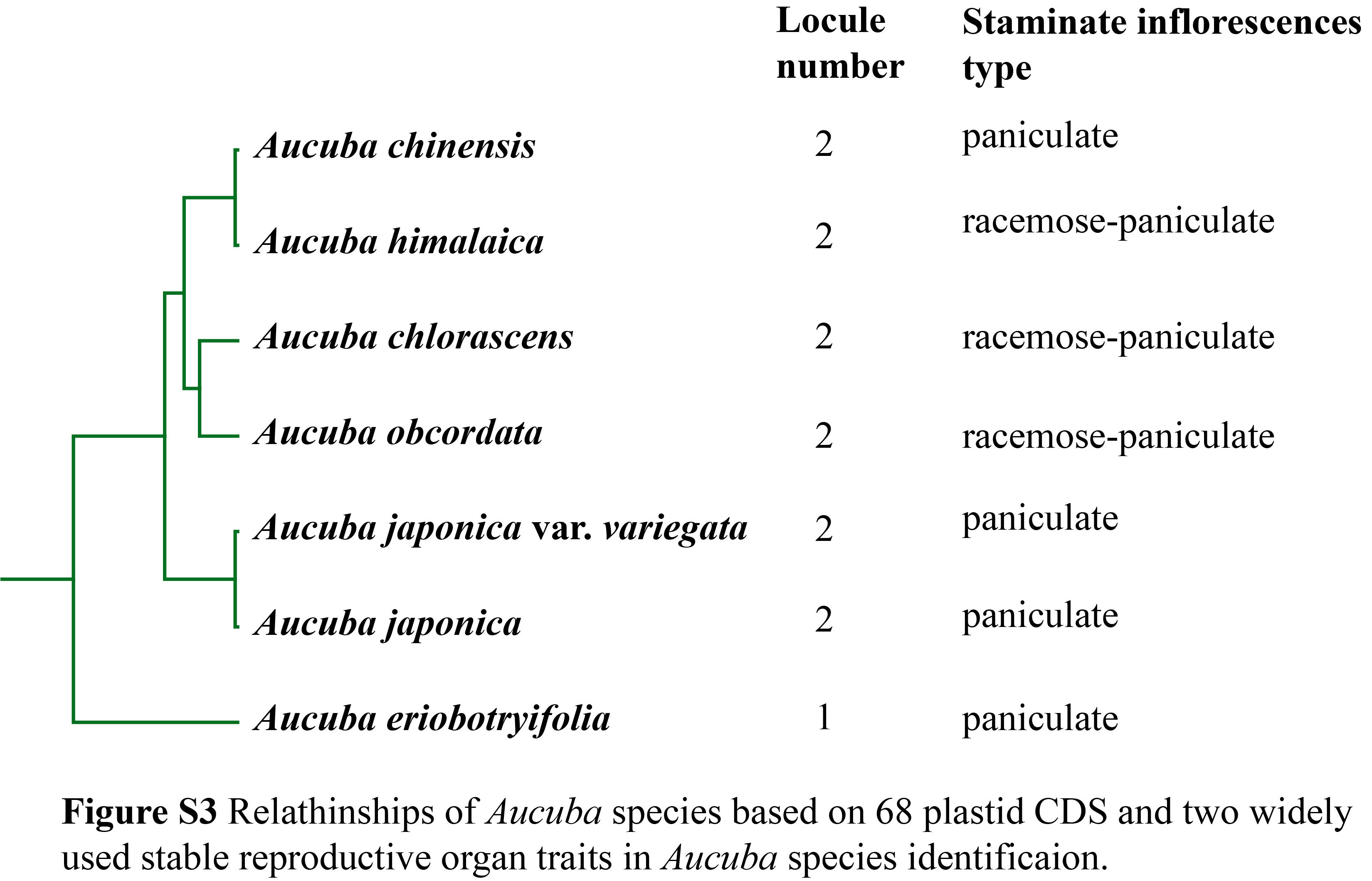

Supplement: Supplementary file 1 [file Image3.JPEG]

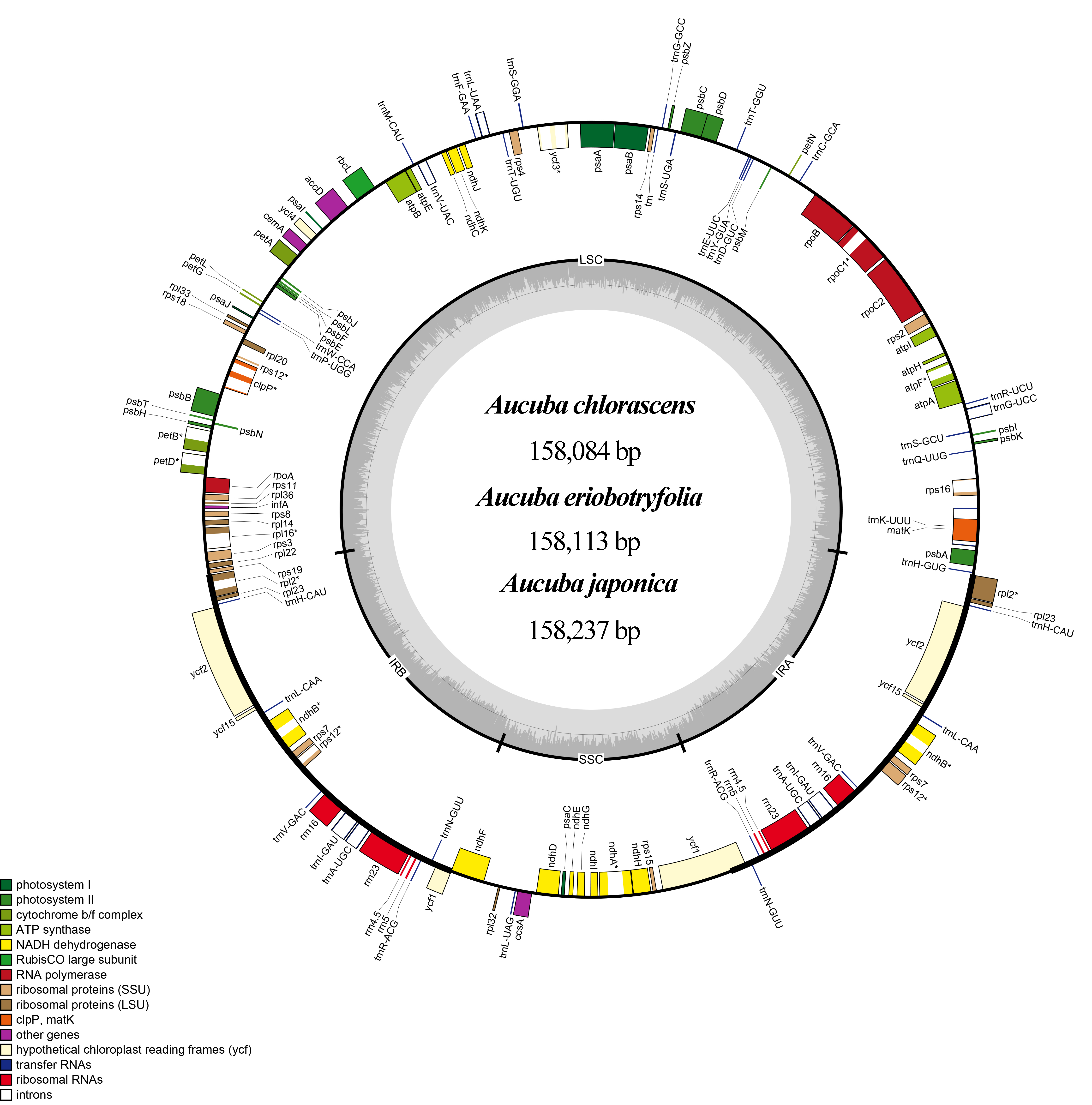

Supplement: Supplementary file 2 [file Image1.JPEG]

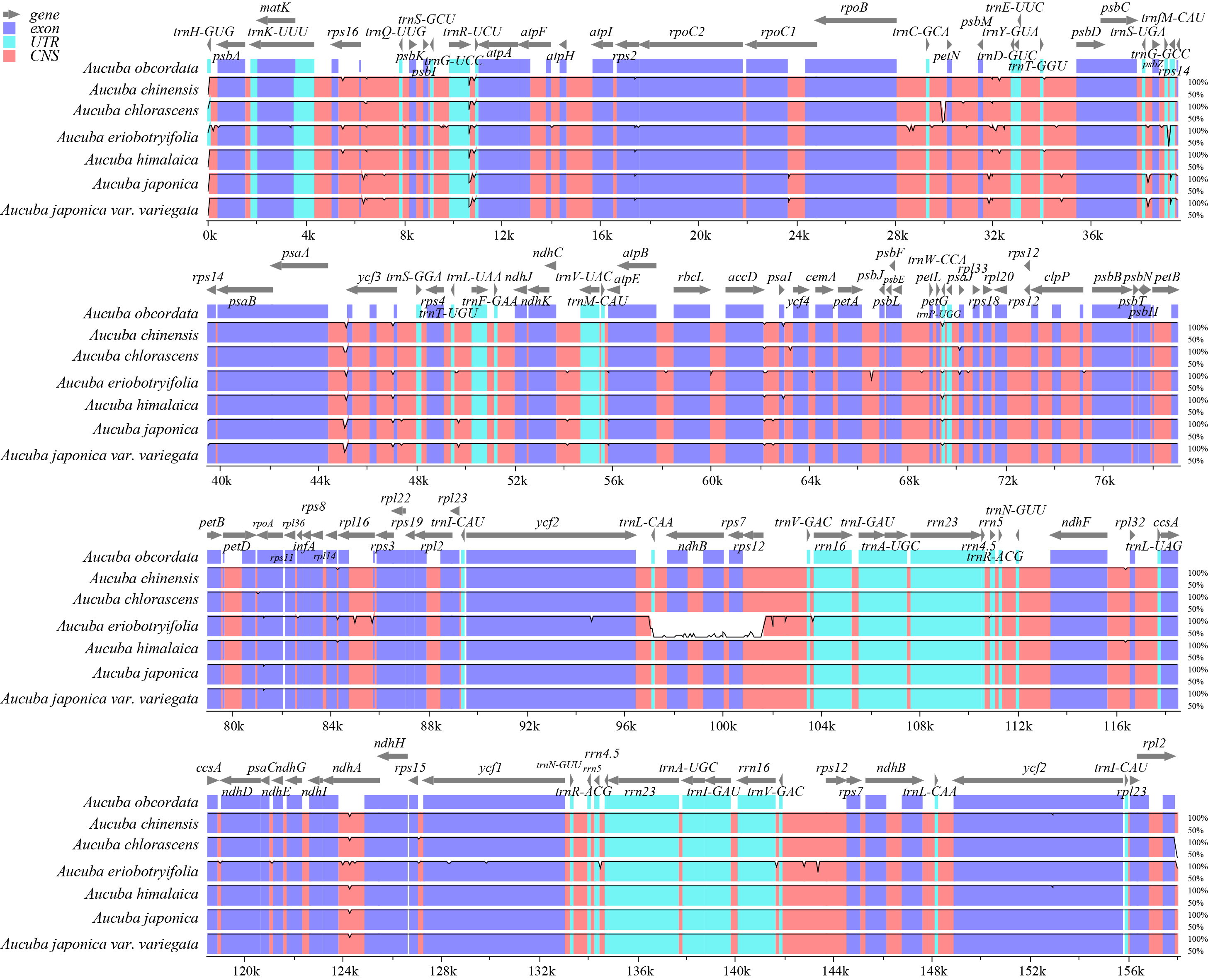

Supplement: Supplementary file 3 [file Image2.JPEG]
